# Spatial structure and organization of the medical device industry urban network in China: evidence from specialized, refined, distinctive, and innovative firms

**DOI:** 10.3389/fpubh.2025.1518327

**Published:** 2025-03-14

**Authors:** Feng Hu, Huijie Yang, Liping Qiu, Shaobin Wei, Hao Hu, Haiyan Zhou

**Affiliations:** ^1^Institute of International Business & Economics Innovation and Governance, Shanghai University of International Business and Economics, Shanghai, China; ^2^International Business School, Shanghai University of International Business and Economics, Shanghai, China; ^3^CEEC Economic and Trade Cooperation Institute, Ningbo University, Ningbo, China; ^4^Institute of Digital Economy and Financial Powerhouse Building, Guangdong University of Finance, Guangzhou, China; ^5^School of Economics, Shanghai University, Shanghai, China; ^6^Graduate School, Nueva Ecija University of Science and Technology, Cabanatuan, Philippines

**Keywords:** specialized and sophisticated firms, urban network, spatial organization, listed firms, medical device industry

## Abstract

**Introduction:**

Investigating the network of firms in a specific industry helps explain industrial location and urban functions and provides guidelines for promoting industrial restructuring and high-quality development.

**Methods:**

This study develops a network model for the relationship between firms and cities based on the data of listed Specialized, Refined, Distinctive, and Innovative (SRDI) medical device manufacturing firms in China to identify the spatial distribution and influencing factors of the urban network of such firms using network analysis and GeoDetector.

**Results and disscusion:**

Three conclusions are obtained from the study. First, the urban network of listed SRDI medical device manufacturing firms exhibits a sparse structure, with the density decreasing from east to west, and the out-degree presenting significant spatial concentration. Suzhou, Shanghai, and Shenzhen are the core of the network power. The in-degree presents low spatial concentration. Clearly differentiated network functions are observed. Second, significant spatial differences are noted between high- and low-level linkage networks from the perspective of corporate governance structure. Third, economic level, labour costs, level of opening-up, talent base, and technological innovation capability have significant effects on the urban network of listed SRDI medical device manufacturing firms.

## 1 Introduction

Medical devices are a key component of the national medical system. Medical device manufacturing firms not only improve therapeutic efficiency and medical service quality by developing and manufacturing medical device, but also play an essential role in military medicine, public health, and other fields (1–4). The Chinese government proposed the concept of Specialized, Refined, Distinctive, and Innovative (SRDI) firms in 2011 and officially promoted them nationwide in 2017, aiming to promote high-quality economic development and industrial restructuring, stimulate innovation in the medical device industry, and enhance international competitiveness. SRDI firms seek to take a leading position in market segments, which, in this study, refer to the medical device industry.

The first to fourth rounds of 9,279 “little giant” SRDI firms announced by the Ministry of Industry and Information Technology of the People’s Republic of China included 514 firms from the medical industry. Of them, 119 are listed firms, and they act as the powerhouse for the high-quality development of the medical device industry in China. By going public, the listed SRDI firms can attract investors and funding sources to increase production capacity and R&D investment to promote long-term development. In addition going public also enhances their social attention, helping them to attract talent (5, 6).

The concept of urban networks was initially analysed from two points of view: abstract inter-city economic linkages in a broad sense and concrete infrastructure networks in a narrow sense (7–10). Specifically, an urban network can be viewed from four different perspectives: (1) the spatial interaction perspective based on the gravity model, (2) the traffic flow perspective based on inter-city aviation, railways, and highways, (3) the information flow perspective based on inter-city population migration, information attention, and social data, and (4) the inter-firm linkage perspective based on inter-firm superior–subordinate and investor relations (11, 12). Of these, the inter-firm linkage perspective has been mainly applied due to firms’ crucial role in urban economic development (13, 14). Previous studies have examined the position of cities in the urban network and the strength of inter-city linkages using data from the APS firm database and the headquarter–branch data of multinational firms (15–23).

In terms of methodology, the chain network model, the affiliation model based on firm ownership, and the partitioned core (city) algorithm are mainly used to create data models to develop an urban network using data on listed firms, top 500 firms, financial firms, and other firms (24–28). Wall and van der Knaap (19) reported a high similarity between the inter-firm network of advanced producer services and that of the whole industry according to data from the world’s 100 largest transnational firms and their branches. Overall, as a result of improved methodologies, an increasing number of data types, and the deepening of perspectives, more realistic characteristics of the urban network linkages have been revealed, which provides useful information on actual urban production and life as well as better guidance for policy development.

In summary, previous studies have provided significant insights into urban networks and the impacts on firm and economic growth. However, few have investigated the spatial organization of SRDI medical device manufacturing firms from the perspective of corporate governance structure. Accordingly, this study aims to fill this gap by exploring the following questions: What is urban network structure of SRDI medical device manufacturing firms in China? Are Beijing, Shanghai, Guangzhou, and Shenzhen at the core of the network as in other urban network studies (29–31)? What factors affect the network? To answer these questions, this study intends to investigate the spatial arrangement of the urban network in China using linkage data of listed SRDI medical device manufacturing firms, their subsidiaries, and sub-subsidiaries from the perspective of corporate governance structure.

The findings will help to identify problems from an alternative perspective, depict the multilateral network relationships among medical device manufacturing firms in China, and promote the efficient allocation of factors among firms. This study will also help bolster research on urban network through the lens of corporate organization, extend research on cities with different industrial network functions, and identify the function of cities in industrial development. Furthermore, by delving into the mechanism of network formation, this study offers recommendations for promoting the SRDI medical device industry, and provides insights into the theoretical research on urban networks.

## 2 Methodology

### 2.1 Subjects

The data for this study came from the Qixin Huiyan big data platform.^1^ A list of listed SRDI medical device manufacturing firms was collected through this platform, and cross-checked against the website of the Ministry of Industry and Information Technology and another data platform, Qichacha.^2^ After excluding firms with abnormal registration status and missing branch office numbers, a total of 15 firms were included, as shown in Table 1. It should be noted that SRDI firms are required to adhere to the rigorous certification standards of “Specialized, Refined, Distinctive, and Innovative” as specified by the Ministry of Industry and Information Technology’s Interim Measures for the Gradual Cultivation and Management of High-Quality Small and Medium-Sized Enterprises. These enterprises lead their specific subfields, and those that are publicly listed demonstrate a high level of industry representativeness.

**Table 1:** Listed SRDI medical device manufacturing firms in China.

NO.	Firm’s name	No.	Firm’s name
1	Andon Health Co., Ltd.	9	Touchstone International Medical Science Co., Ltd.
2	Sansure Biotech Inc.	10	Chison Medical Technologies Co., Ltd.
3	Shanghai Henlius Biotech Co., Ltd.	11	Apt Medical Inc.
4	Improve Medical Instruments Co., Ltd	12	Honsun (Nantong) Co., Ltd
5	Sharetronic Data Technology Co., Ltd.	13	Ave Science & Technology Co., Ltd.
6	Shanghai Aohua Photoelectricity Endoscope Co., Ltd.	14	Hob Biotech Group Corp., Ltd.
7	Shenzhen Glory Medical Co., Ltd.	15	Kontour (Xi’an) Medical Technology Co., Ltd.
8	Suzhou Iron Technology Co., Ltd.		

Furthermore, relevant data, included fields such as the company name, unified social credit code, registration address, and establishment date, of these firms along with their subsidiaries and sub-subsidiaries were collected using the firm genealogy section of the Qixin Huiyan platform. During the data cleaning phase, the address information was standardized using regular expressions, and the comprehensive relationships were further validated by adopting the equity penetration mapping from the Qichacha platform. As a result, 746 subsidiaries and 1805 sub-subsidiaries were included after data cleaning and processing.

In the network relationship modeling phase, a directed weighted graph G = (V,E) is constructed, where the node set V comprises 2,551 corporate entities, and the edge set E represents control relationships. For example, when listed SRDI medical device manufacturing firms A, B, and C have subsidiaries D, E, and F, subsidiaries G and H, and subsidiary I, respectively, and subsidiaries D and H have sub-subsidiaries J and K and sub-subsidiaries L, M, and N, respectively, the network linkages are (AD + AE + AF + BG + BH + CI) + (DJ + DK + HL + HM + HL). Network linkages in the cities where the firms are registered can then be established. Finally, a data matrix is constructed from the headquarter–branch relationship, and spatial network visualization is carried out using Gephi and ArcGIS.

### 2.2 Methods and data

#### 2.2.1 Social network analysis

To identify the urban network characteristics of the listed SRDI medical device manufacturing firms, the changes in centrality, density, clustering coefficient, and average path length of the urban network were analysed through social network analysis using Gephi software (32–36).

#### 2.2.2 GeoDetector

The city-weighted centrality and the proportions explained by production factors, technological innovation capability, financial development, and other influencing factors in the urban network of listed SRDI medical device manufacturing firms were determined by formulas proposed by Zhou et al. (37), Zhu et al. (38), Li et al. (39) using the factor detector in GeoDetector.

## 3 Results

### 3.1 Network structural characteristics

The primacy and 10-city indices of the network out-degree are calculated to be 0.307 and 0.348, respectively, indicating very significant spatial concentration. Most cities have a low out-degree. Because listed SRDI medical device manufacturing firms were mainly located in Suzhou and Shenzhen, these two cities are dominant in the network. Xi’an, Shanghai, Hangzhou, and Guangzhou also have dominance over network resources because they are the main locations of subsidiaries. All other eastern and central cities have a low out-degree, and 77 of them have an out-degree of zero, indicating that they are edge cities in the network power.

The primacy and 10-city indices of the network betweenness are 0.183 and 0.208, respectively. The top ten cities account for 87.78% of the total betweenness. Suzhou, Shanghai, and Shenzhen, each accounting for more than 10% of the total betweenness, are the core of the network power. Meanwhile, Hangzhou, Beijing, Guangzhou, Nanjing, and Chengdu are important bridging centres for network power. Furthermore, Hefei, Xi’an, Changsha, Jiaxing, and Wuhan, despite having low betweenness, are able to bridge cities and thus act as secondary bridging points for the network power. Conversely, Anqing, Foshan, Lishui, Taiyuan, Dalian, and Ma’anshan have a betweenness value of zero, indicating that they are edge cities in the network power bridging system.

The primacy and 10-city indices of the network in-degree are 0.093 and 0.158, respectively, compared to the out-degree and betweenness centrality, indicating low spatial concentration. The top ten cities in terms of in-degree are all provincial capitals and municipalities directly under the central government. The cities ranked 10th to 20th, including Zhuhai, Wuxi, Hefei, Tianjin, Jiaxing, Chongqing, Nanchang, Ningbo, Changzhou, and Xiamen, are all relatively economically developed cities. These findings demonstrate the strong economic dependence of the listed SRDI medical device manufacturing firms in the spatial layout of their subsidiaries and sub-subsidiaries.

**Figure 1:**
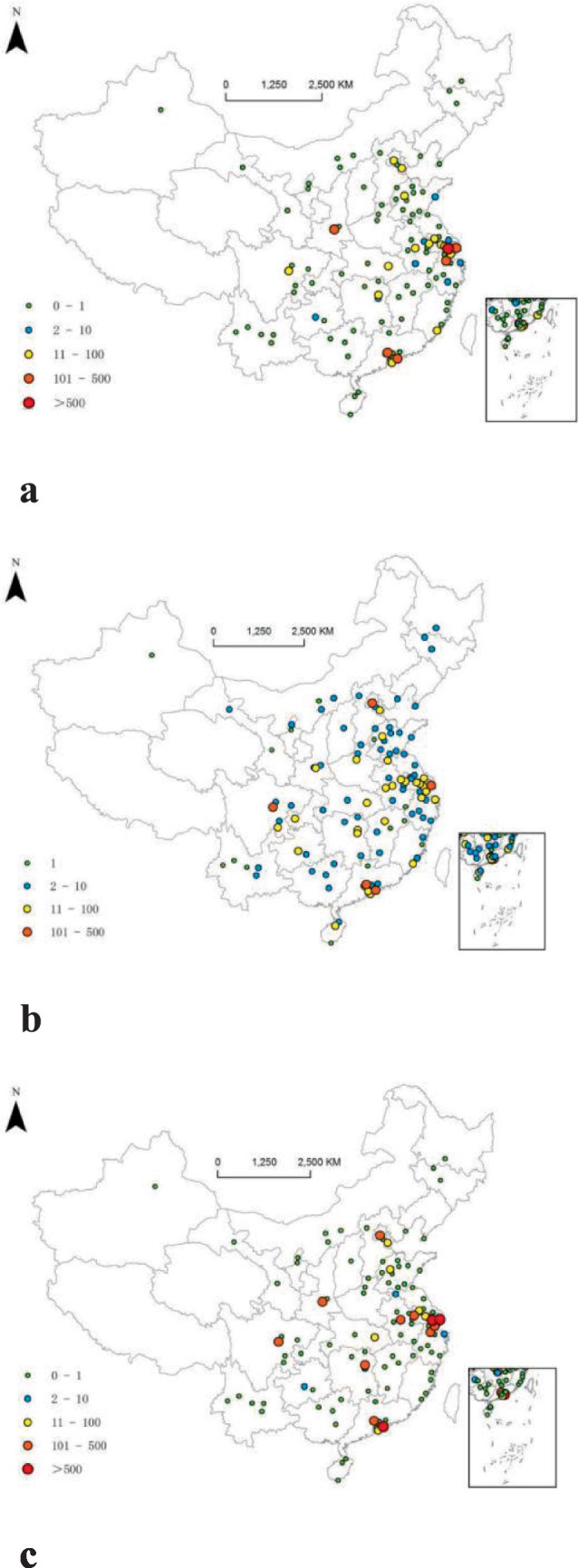
Spatial distributions of out-degree, in-degree, and betweenness in the urban network of listed SRDI medical device manufacturing firms. **(a)** Out-degree. **(b)** In-degree. **(c)** Betweenness.

In general, cities with high out-degree, betweenness, and in-degree are concentrated in the eastern coastal areas. The urban network of listed SRDI medical device manufacturing firms is highly affected by regional economic development, education, and scientific research, which is similar to previous reports (40, 41) (see Figure 1).

### 3.2 Spatial differentiation of network functions

**Figure 2:**
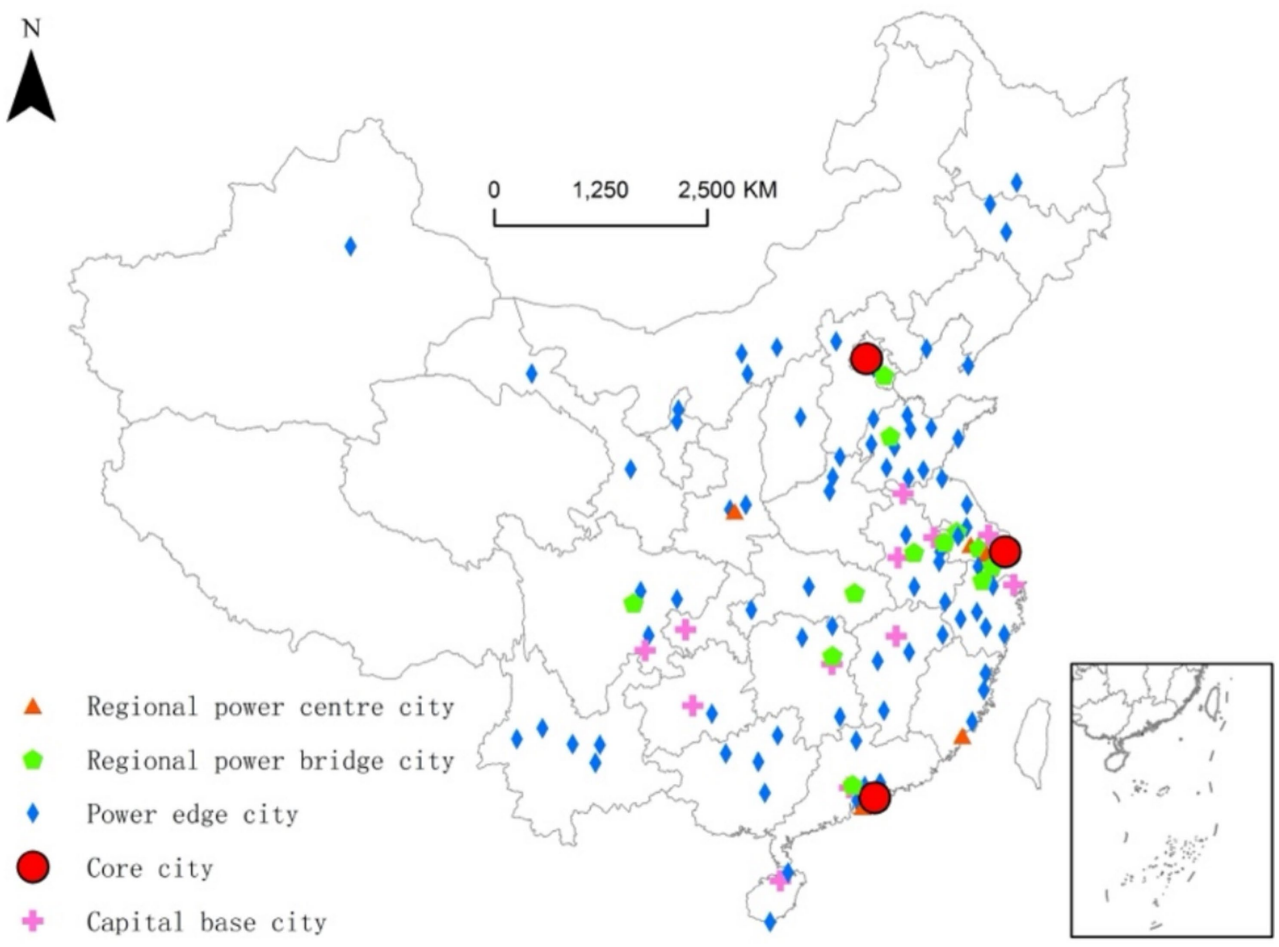
Spatial distributions of network functions in the urban network of listed SRDI medical device manufacturing firms.

By measuring the out-degree, in-degree, and betweenness, the cities are classified into five categories according to network functions as shown in Figure 2. Core cities are defined as those ranked among the top five in terms of all three indices. Suzhou, Shanghai, and Shenzhen rank in the top three in terms of betweenness centrality, with their combined share exceeding 50%. Additionally, these cities rank in the top four based on out-degree, and their combined share also exceeds 50%. They are thus core cities in the power system of the SRDI medical device industry. Suzhou, Zhuhai, Changzhou, Xiamen, and Xi’an are among the top 20 in out-degree and have an out-degree higher than betweenness. This makes them the regional power centre cities in the SRDI medical device industry. Regional power bridge cities, defined as being among the top 20 in betweenness and having betweenness higher than out-degree, include Guangzhou, Chengdu, Nanjing, Wuhan, Hangzhou, Changsha, Wuxi, Hefei, Tianjin, Jiaxing, Jinan, and Yangzhou. These cities can thus be classified as regional bridges in the power system of the SRDI medical device industry. Capital base cities, defined as being among the top 30 in in-degree and ranked below 20th place in both out-degree and betweenness, include Chongqing, Nanchang, Ningbo, Chengmai, Xuzhou, Chuzhou, Xiangtan, Nantong, Foshan, Yibin, Lu’an, and Guiyang. They mainly serve as capital-gathering centres in the network. The remaining cities are edge cities in the power system.

### 3.3 Network linkages

**Figure 3:**
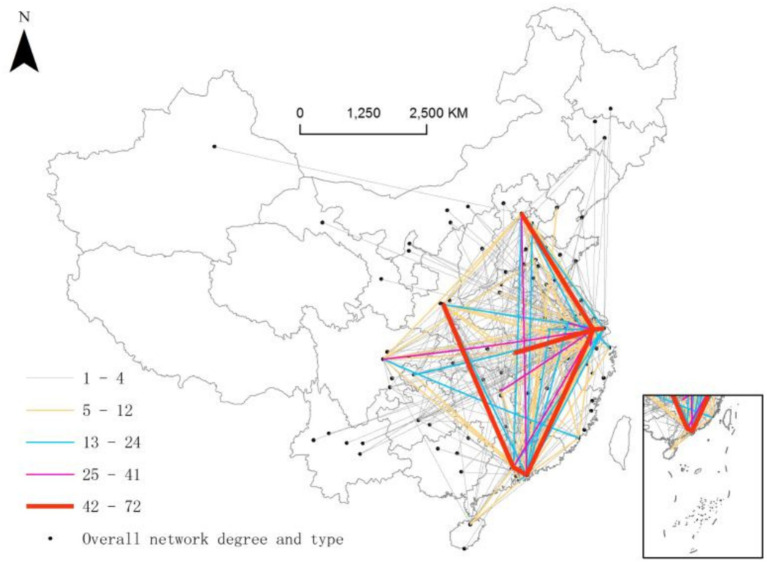
Urban network of listed SRDI medical device manufacturing firms.

Using UCINET software, the urban network density of listed SRDI medical device manufacturing firms is calculated to be 0.042, a gap as large as 0.958 from the saturated density. The number of network edges is 492, which is also much lower than the highest theoretical edge number of 11,772. These findings suggest that the overall network has a sparse structure and is in the development stage. At the current stage, maintaining an optimal distance between nodes within a sparse network can preserve their distinctiveness and expertise, while also creating “structural holes” that facilitate cross-boundary innovation and yield competitive advantages. Nevertheless, excessive sparsity may diminish opportunities for complementary benefits across regions, thereby impeding the formation of synergistic effects and hindering the overall development of the innovation environment. In terms of spatial distribution (Figure 2), the network density decreases from east to west. This spatial distribution characteristic reflects the regional disparities in the medical device industry. Specifically, the eastern regions exhibit higher levels of innovation and development density, with high-level edges clearly centered around Suzhou. Suzhou serves as the core for most of these high-level edges (75%), including nine first- and second-level edges, which, in order of weight, connect Suzhou to Beijing, Shenzhen, Shanghai, Wuhan, Changsha, Chengdu, Nanjing, Hangzhou, and Guangzhou, respectively. Thus, it can be concluded that Suzhou plays a supporting and driving role in the urban network of listed SRDI medical device manufacturing firms. Other high-level edges include Shenzhen and Guangzhou, Xi’an and Guangzhou, and Shenzhen and Beijing, representing linkages in the eastern, central, and western regions, respectively (see Figure 3).

## 4 Discussion

According to the hierarchy of parent firm > subsidiary > sub-subsidiary, two types of networks are constructed based on corporate governance structure: a high-level inter-firm network between cities where parent firms and subsidiaries are located (network A), and a low-level inter-firm network between cities where subsidiaries and sub-subsidiaries are located (network B). In this way, the spatial distribution of the urban network of listed SRDI medical device manufacturing firms is presented at the city level in a comprehensive multi-view manner.

### 4.1 Network topological features based on corporate governance structure

Networks A and B have a density of 0.028 and 0.04, with 79 and 105 nodes, respectively. Overall, the urban network of listed SRDI medical device manufacturing firm ownership has very weak linkages. There remains substantial room for improvement of factor flows between urban nodes. The density of network B is higher than that of network A, indicating that lower-level firms are more closely connected. The average path length of networks A and B is 2.078 and 2.238, respectively, and only a few strong direct connections exist between urban nodes.

In general, two or more intermediary cities are needed to establish a connection. Shanghai, Suzhou, and Shenzhen lead both networks in terms of betweenness. This suggests that these three cities have high control over the diffusion and transfer of investment resources of the listed SRDI medical device manufacturing firms. In addition, Guangzhou, Nanjing, Chengdu, Hefei, Jiaxing, and Changsha are at the second level. As regional economic centres and transportation hubs, these cities serve as important bridges in the network by virtue of their close connections with Shenzhen, Shanghai, and Suzhou. Most cities, including Urumqi, Shaoguan, Lanzhou, and Jingmen, are at the third level. These local cities play a vital supporting role in the urban network of listed SRDI medical device manufacturing firms. In general, provincial capitals and municipalities directly under the central government in the eastern coastal region have high betweenness and great control over the concentration and diffusion of resources (see Table 2).

**Table 2:** Ranking of cities by betweenness in the two networks.

Level	Network A	Network B
First level	Suzhou, Shanghai, Shenzhen, and Xi’an	Suzhou, Shanghai, Shenzhen, Hangzhou, and Beijing
Second level	Changsha	Guangzhou, Nanjing, Chengdu, Hefei, Jiaxing, Changsha, Wuhan, and Tianjin
Third level	Taiyuan, Changchun, Zhenjiang, Tianjin, Zhuhai, and Wuxi, etc.	Jinan, Yangzhou, Wuxi, Changzhou, Zhuhai, Qingdao, and Guiyang, etc.

Cities at each level in the two networks are ranked by betweenness.

### 4.2 Network linkages based on corporate governance structure

In network A, in terms of linkage strength, investment in Guangzhou by Xi’an generates the most linkages, being at the first level. The linkages of investment in Changsha by Suzhou are at the second level. The third-level linkages occur between Suzhou, Xi’an, Shanghai, Shenzhen, Beijing, Nanjing, Chengdu, Hefei, and Tianjin, all due to the driving role of Suzhou, Xi’an, Shanghai, and Shenzhen. In terms of centrality, no cities have a centrality higher than 500. Only one city, Suzhou, has centrality between 301 and 500, and three cities, Shenzhen, Xi’an, and Shanghai, have centrality between 101 and 300.

In network B, in terms of linkage intensity, investment in Beijing by Suzhou and in Guangzhou by Shenzhen generate the most linkages, being at the first level. The linkages of investment in Wuhan, Shanghai, Shenzhen, and Hangzhou by Suzhou are at the second level. Third-level linkages occur between 13 cities, including Xiamen, Beijing, Wuhan, and Guangzhou. In terms of centrality, only one city, Suzhou, has centrality higher than 500; Shenzhen, Guangzhou, and Shanghai are at the second level; and Hangzhou, Beijing, Chengdu, Wuhan, and Nanjing are at the third level.

**Figure 4:**
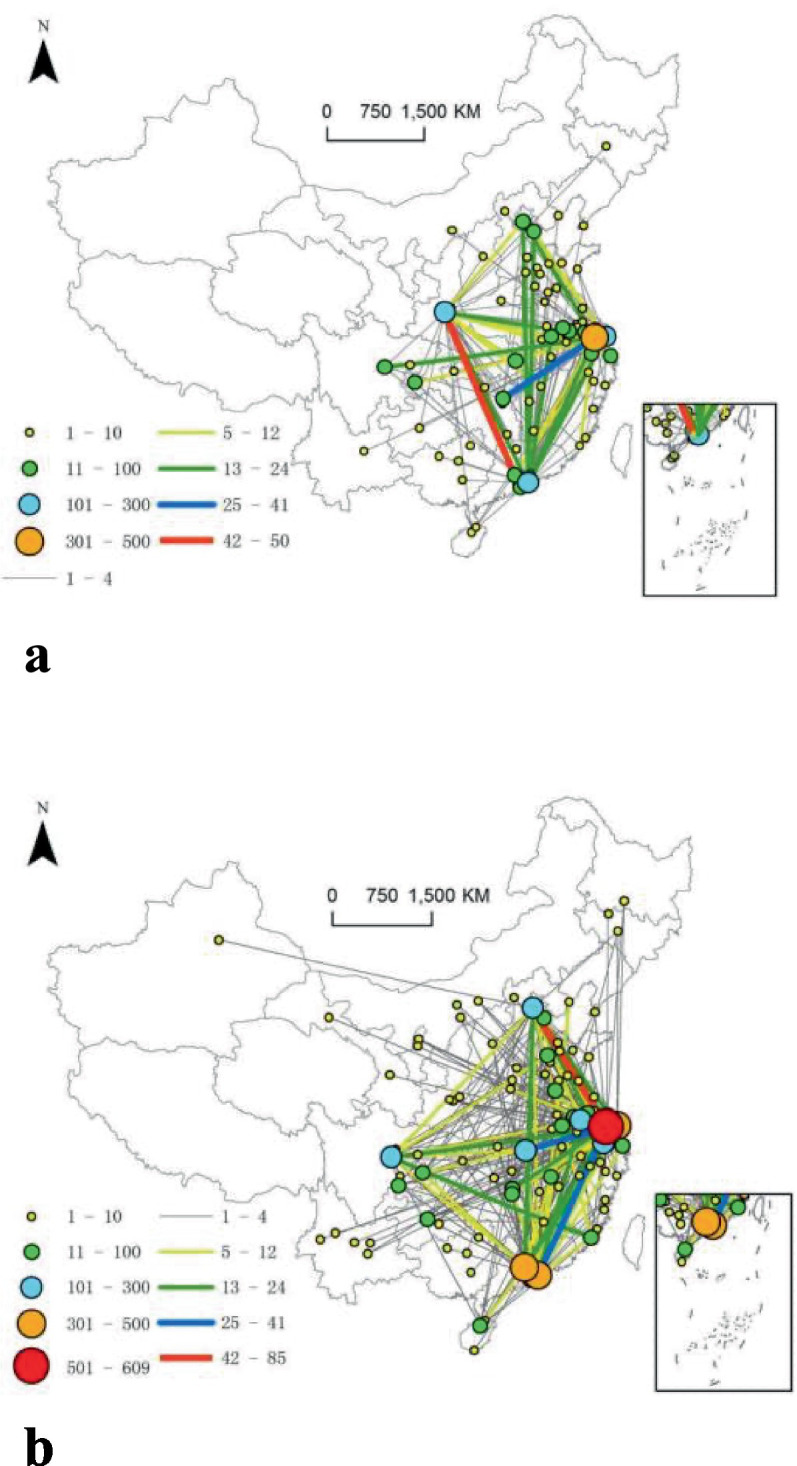
Network linkages based on corporate governance structure. **(a)** Parent–subsidiary network and centrality. **(b)** Subsidiary–sub-subsidiary network and centrality.

According to the findings, it can clearly be seen that high-strength linkages mostly occur between cities with high centrality, presenting significant rich-club features. In other words, the network exhibits obvious hierarchical characteristics. The low-level inter-firm (subsidiaries/sub-subsidiaries) linkages in the corporate governance structure in network B are decentralized and intertwined. Not all closely linked cities in the network are geographically adjacent, and economic development is the most important influencing factor (see Figure 4).

### 4.3 Network overlay based on corporate governance structure

Networks A and B are overlaid to facilitate a more in-depth analysis of the differences in their network structures. The results show that only 95 out of 569 pairs of linked cities have both parent–subsidiary and subsidiary–sub-subsidiary linkages. Compared with high-level ones, low-level inter-firm linkages are extensive and close. Both adjacent and distant links exist in the urban network of listed SRDI medical device manufacturing firms. Short-range links are common within a province, whereas distant links mainly occur between provinces. The intertwining of the two modes of links shows a noticeable flattening trend.

**Figure 5:**
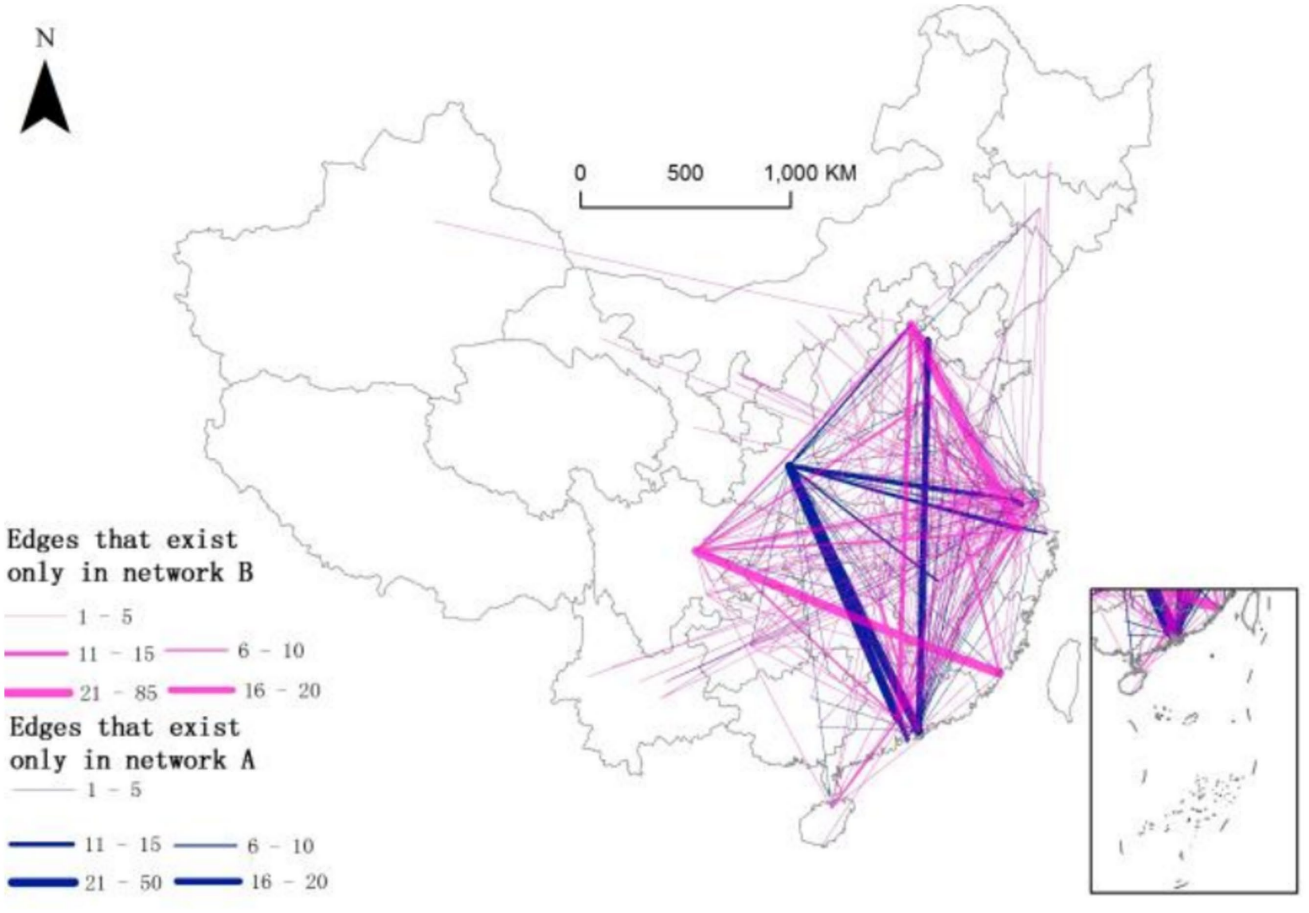
Overlay of networks A and B.

In addition, when distinguished by corporate governance structure, different characteristics emerge about the linkages of the urban network of listed SRDI medical device manufacturing firms. For example, Xi’an is closely connected with the central cities in the Pearl River Delta through the investment linkages of the listed SRDI medical device manufacturing parent firms. Guangzhou and Hangzhou play a leading role among edge cities in the central and western regions through the investment linkages of subsidiaries, thereby optimizing the urban network structure of listed SRDI medical device manufacturing firms (see Figure 5).

### 4.4 Factors influencing the network

Considering comprehensiveness and data availability, and inspired by previous studies, e.g., Duranton and Puga (42), Nabelsi and Gagnon (43), Li et al. (44), Hu et al. (45), the weighted network centrality is used as the dependent variable, and GDP per capita representing the economic level, average salary of urban non-private-sector employees representing the labour costs, total imports and exports representing level of opening-up, number of undergraduates and college students representing the talent base, number of patents granted representing technological innovation capability, and administrative level of the city representing political resources are used as independent variables. The correlation coefficients between the weighted centrality of the urban network and the influencing factors were determined using GeoDetector after all variables were stratified into five groups by natural breaks using ArcGIS. In particular, in terms of administrative level, Beijing takes the value 5, Shanghai, Chongqing, and Tianjin take 4, provincial capitals take 3, sub-provincial cities take 2, and general cities take 1. Data on influencing factors were all sourced from *China City Statistical Yearbook 2022*.

GeoDetector analysis reveals that all factors, except political resources, have a significant influence on the weighted centrality of the urban network. It suggests that the urban network’s weighted centrality is influenced by economic level, labour costs, level of opening-up, talent base, and technological innovation capability. Individually, these five influencing factors explain from 19 to 74% of the results and are categorized into core and secondary influencing factors by the proportion explained. Specifically, the core influencing factors are level of opening-up and technological innovation capability; and the secondary ones are economic level, labour costs, and talent base.

The correlation with level of opening-up and technological innovation capability ranks first and second, respectively, in both the urban network of listed SRDI medical device manufacturing firms and networks A and B. Specifically, technological innovation capability is the key to the rapid and high-quality development of high-tech industries like the medical device industry (46–48). Open cities attract international capital and facilitate exports and financing (49, 50). Regression analysis of corporate governance structure reveals the highest correlation with level of opening-up for high-level inter-firm linkages (network A). This may be due to the greater demand of the listed SRDI medical device manufacturing firms for international capital and financing. Low-level inter-firm linkages (network B) show the highest correlation with technological innovation capability. Data analysis reveals that more than 60% of the sub-subsidiaries are technology or laboratory firms, which attach more importance to local technological innovation capability when selecting locations.

The correlation with labour costs, economic level, and talent base ranks third to fifth, respectively. Labour costs are positively related to the quality of human resources, and professional talent from colleges and universities are at the core of the medical device industry (51, 52). In addition, a high economic level substantially promotes the development of local medical device industry (53, 54). Regression analysis of corporate governance structure reveals higher correlation with labour costs, economic level, and talent base for low-level inter-firm linkages (network B).

No significant correlation is found with political resources in the urban network of listed SRDI medical device manufacturing firms. Although a high administrative level facilitates the collection of political resources by firms, it has a relatively small influence and is not a key factor in the location selection of these firms. One possible reason for the absence of localized advantages in the medical device industry is its predominantly national policy framework, which limits companies’ ability to secure preferential treatment through regional political connections. For example, the Chinese government has implemented nationwide policies to foster innovation in the sector, including the “14th Five-Year Plan for Biological Economy Development” and the medical device registrant system. These policies are not confined to high-administrative-level cities; rather, they are designed to benefit the entire industry, thereby offering companies in various locations equal access to policy advantages. Consequently, the impact of different administrative levels is diminished (see Table 3).

**Table 3:** Regression analysis in GeoDetector.

Influencing factor	Measure	Urban network of listed SRDI medical device manufacturing firms		Network A		Network B	
		Q value	Significance	Q value	Significance	Q value	Significance
Economic level	GDP per capita	0.4270	0.000	0.3915	0.000	0.4415	0.000
Labour costs	Average salary of urban non-private sector employees	0.5074	0.000	0.4093	0.000	0.5089	0.000
Level of opening-up	Total imports and exports	0.6386	0.000	0.6647	0.000	0.6275	0.000
Talent base	Number of undergraduates and college students	0.3824	0.000	0.2370	0.009	0.3016	0.003
Technological innovation capability	Number of patents granted	0.7244	0.000	0.6913	0.000	0.7369	0.000
Political resources	Administrative level of the city	0.3060	0.157	0.1927	0.597	0.2400	0.362

## 5 Conclusion

This study has developed a network model for the relationship between firms and cities based on the data of listed SRDI medical device manufacturing firms to identify the spatial distribution and influencing factors of the urban network of such firms in China using network analysis and GeoDetector. The following conclusions are obtained.

First, the urban network of listed SRDI medical device manufacturing firms has a sparse structure, with the density decreasing from east to west. Compared to the western regions, the eastern regions such as Shanghai, Suzhou, and Shenzhen have developed economies and abundant scientific and technological resources. These areas demonstrate industrial agglomeration and frequent inter-firm communication, resulting in higher network density. Suzhou is the core of most high-level edges, and only a few cities have a high out-degree and betweenness. These two indices present significant spatial concentration. The opposite is true for in-degree, and distinct network functions are observed. Accordingly, cities can be divided into core, power centre, power bridge, capital base, and power edge cities.

Second, both the parent–subsidiary urban network A and the subsidiary–sub-subsidiary urban network B constructed based on corporate governance structure have a sparse structure. Provincial capitals and municipalities directly under the central government in the eastern coastal region have high betweenness and great control over the concentration and diffusion of resources. High-strength linkages mostly occur between cities with high centrality, presenting significant rich-club features. In addition, the network exhibits noticeable hierarchical characteristics. Compared with high-level ones, low-level inter-firm linkages are extensive and close.

Lastly, the GeoDetector results reveal that all factors, except political resources, significantly influence the weighted centrality of the urban network. This finding suggests that the weighted centrality of the urban network is influenced by economic level, labour costs, openness to the outside world, talent base, and technological innovation capability.

### 5.1 Implications

First, guide the development of innovative industrial clusters. According to the study findings, the core nodes of the urban network of listed SRDI medical device manufacturing firms are concentrated in provincial capitals and municipalities directly under the central government, including Shenzhen, Xi’an, Shanghai, Hangzhou, and Guangzhou, as well as Suzhou, a city with industrial advantages. When designing strategies for developing the medical device industry, the Chinese government should promote strategic exchanges in the medical device industry between core cities while taking different regional advantages into account based on the network functions of each city. It is recommended to prioritize support for core cities in establishing technological innovation platforms and industrial clusters, thereby strengthening internal networks and reinforcing their leadership positions within the industry.

Second, establish cross-regional collaboration platforms. To address the challenges of low network density and limited inter-node connections, the government should assume a pivotal role in fostering regional or industry-specific technological alliances, information-sharing platforms, and collaborative innovation networks. Organizing regular technical seminars, joint training programs, and activities that bring together industry, academia, and research institutions can strategically align non-core regions with “hub” cities. Facilitating cross-regional technology transfer, joint research and development, and resource sharing can streamline the information flow, resulting in an innovation network with multiple interconnected nodes. Encouraging close collaboration between core cities and surrounding small to medium-sized cities will unlock the overall potential for collaborative innovation within the industry.

Third, pay attention to regional talent bases. It is important for nodes in the urban network of listed SRDI medical device manufacturing firms to nurture the local talent base. Specifically, efforts should be made to introduce relevant talents, enhance training mechanisms, and increase fund investment in education, especially higher education, thereby improving regional innovation and construction of knowledge networks in medical device manufacturing. In addition, regional cities should aim to enhance innovative exchanges and cooperation with core cities, such as Shenzhen and Xi’an, so as to improve the regional ability to absorb and transform medical device manufacturing knowledge.

### 5.2 Theoretical implications

First, this study investigates the position and role of each node city in the urban network of listed SRDI medical device manufacturing firms. By identifying the positions and roles of different cities within the network, this study enhances the understanding of urban economic geography at the provincial and municipal levels (13, 15). Traditional urban network research primarily focuses on infrastructure and administrative hierarchies; however, this study expands the field by revealing how businesses shape urban interactions and integrating industrial cluster theory with inter-city economic relations (10). This effort has not only helped to identify the industrial development functions and roles of different cities, but also advances theoretical research on provincial and municipal geography.

Second, the urban network of listed SRDI medical device manufacturing firms is analysed by social network analysis from the perspective of corporate governance structure, thereby extending and bolstering research on urban networks from the perspective of corporate organization. Unlike previous studies that emphasized transportation and administrative connections (11, 12), this research highlights the impact of ownership and control structures on the connectivity of urban networks. It provides a new perspective on how the SRDI firms can act as agents in shaping urban networks.

Lastly, this study analyses the urban network using data from medical device manufacturing firms based on industrial economics, which helps to extend research on cities with different industrial network functions and provides guidance for the development of the medical device industry. Additionally, the research challenges the perspective that cities with high administrative levels automatically attract businesses (55). It emphasizes the crucial role of a comprehensive business environment in enterprise location decisions, offering a new analytical framework for city-industry interaction.

### 5.3 Limitations

This study has investigated the characteristics and influencing factors of the urban network of listed SRDI medical device manufacturing firms in China. However, due to space limitations and the specific scope of the research topic of this paper, further research is required in three areas:

First, this study regarded subsidiaries and sub-subsidiaries as equally important in the construction of the urban network based on the corporate headquarter–branch relationship. However, great differences may exist between them in practical business management, and further work can be undertaken to consider this issue.

Second, the branches of listed SRDI medical device manufacturing firms were not classified by industry attributes. However, there are great differences in urban network linkages among branches with different industry attributes, such as research centre and sales promotion. Future research could utilize business registration data to conduct a more detailed classification of branch office functions, further deepening the understanding of the interaction mechanisms between micro-level organizational structures of enterprises and urban networks.

Lastly, the mechanism of network evolution could be expanded based on this study. For example, regional industrial policies could be quantified based on the level of policy implementation at the provincial, municipal, or district levels and their frequency. Additionally, transportation location could be quantified based on the distance from provincial or municipal government centers.

## Data Availability

The original contributions presented in the study are included in the article/supplementary material, further inquiries can be directed to the corresponding authors.

## Footnotes

^1^ https://b.qixin.com/

^2^ https://www.qcc.com/
